# Admixture and Selection Driven by El Niño–Southern Oscillation Events Shape the Genetic Structure of *Octopus mimus*–*O. hubbsorum* Complex Across the Humboldt and South Equatorial Current Transition Zone

**DOI:** 10.1002/ece3.74222

**Published:** 2026-09-02

**Authors:** Jackeline Ruiz‐Mendoza, Vanessa C. Bieker, Jaime G. Morin‐Lagos, Franz Cardoso, Ximena Vélez‐Zuazo, Joanna Alfaro‐Shigueto, Marcial Arellano‐Martínez, Raquel Siccha‐Ramirez, Michael D. Martin, Jorge L. Ramirez

**Affiliations:** ^1^ Facultad de Ciencias Biológicas Universidad Nacional Mayor de San Marcos Lima Peru; ^2^ Department of Natural History NTNU University Museum, Norwegian University of Science and Technology Trondheim Norway; ^3^ Royal Botanic Gardens, Kew Richmond, Surrey UK; ^4^ Center for Conservation and Sustainability, Smithsonian National Zoological Park and Conservation Biology Institute Washington DC USA; ^5^ Grupo de Investigación ‘Soluciones para la Biodiversidad’, Carrera de Biología Marina Universidad Científica del Sur Lima Peru; ^6^ Instituto Politécnico Nacional, Centro Interdisciplinario de Ciencias Marinas La Paz Baja California Sur Mexico

**Keywords:** gene flow, hybridization, oceanographic barriers, secondary contact

## Abstract

Marine transition zones, where contrasting water masses converge, can function as natural laboratories for studying admixture and early stages of speciation. The genomic structure of the eastern Pacific *Octopus mimus* — *O. hubbsorum* complex was investigated by analyzing whole‐genome sequencing data from 67 individuals sampled along the west coast of the Americas, spanning Mexico and the Peruvian coast. This includes the South Equatorial Current, the transition zone, and the Humboldt Current System. The mitochondrial genomes fell into two major genetic clades that largely corresponded to the warm‐water northern ( *O. hubbsorum* ) and cold‐water southern ( *O. mimus* ) lineages. Analyses of the nuclear genomes revealed the same bipartite structure but also identified a broad admixture zone characterized by two different admixed clades (Admixed‐Cold and Admixed‐Warm). The results suggest that episodic relaxation of oceanographic barriers during El Niño–Southern Oscillation (ENSO) events promotes secondary contact and gene flow, resulting in admixed individuals recurrently during ENSO years. However, the survival of these admixed individuals depends on the adaptive genetic composition of each organism and the prevailing environmental conditions. Outlier SNP analysis supports these findings, where the Admixed‐Cold cluster shares mainly the adaptive genetic component identified as outliers in *O. mimus* , while Admixed‐Warm is linked to those in *O. hubbsorum* . The *O. mimus–O. hubbsorum* complex is currently occupying a gray zone of speciation, in which selection and climate‐driven connectivity act in tandem to shape genomic divergence.

## Graphical abstract

Whole‐genome sequencing of the *Octopus mimus* – *O. hubbsorum* complex in the eastern Pacific identified two main genetic clades—northern warm‐water and southern cold‐water lineages—with a broad admixture zone during ENSO years where admixed individuals reflect both their genomic makeup and local environments.

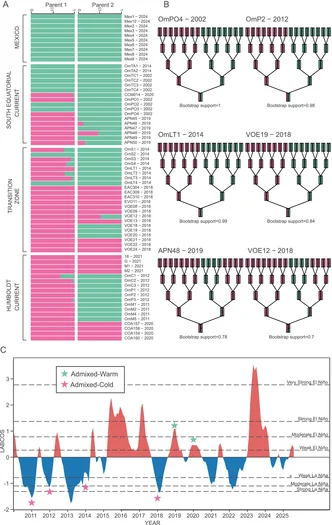

## 1 Introduction

**FIGURE 1:**
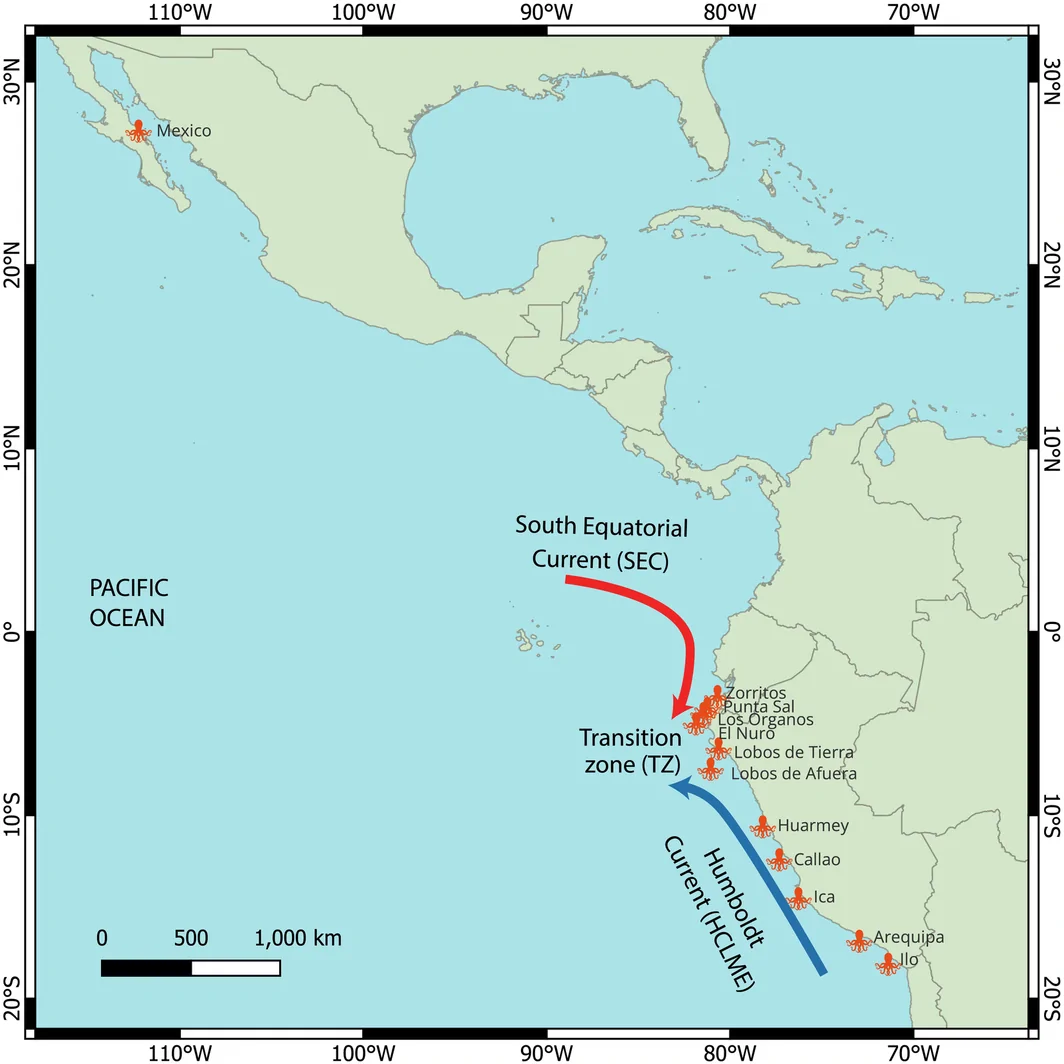
Sampling locations along the Peruvian and Mexican coast in San Bruno. Sampling sites include Zorritos, Punta Sal, El Ñuro and Los Órganos (SEC), Sechura and Islas Lobos de Afuera, Lobos de Tierra (TZ), and Huarmey, Callao, Ica, Arequipa (Matarani) and Ilo (HCLME).

Oceanographic processes exert profound influences on the genetic diversity and population dynamics of marine organisms (López‐Márquez et al. 2021; Zeng et al. 2020). These processes, including ocean currents, temperature gradients, and nutrient availability, play a critical role in shaping the distribution, connectivity, and adaptation of marine species (Geng et al. 2021; Li et al. 2021; Vera et al. 2023; White et al. 2010). In the Eastern Southeast Pacific, the Peruvian coast stands out as a highly dynamic marine environment, where two major ocean currents converge, influencing biodiversity and species dispersal patterns. The Humboldt Current Large Marine Ecosystem (HCLME), which extends from central Chile to northern Peru, has a significant impact on the region by transporting cold and nutrient‐rich waters and supporting a rich biodiversity (Montecino and Lange 2009). In contrast, the South Equatorial Current (SEC) transports warm surface waters from the Ecuadorian coast westward into the Pacific Ocean, influencing regional weather patterns and supporting diverse marine life along its path (Montes et al. 2010). These two contrasting water currents meet along the northern Peruvian coast, creating a transition zone (Ibanez‐Erquiaga et al. 2018; Montecino and Lange 2009) between Cabo Blanco (4°15′S) and Punta Aguja (5°47′S) (Figure 1) (Hooker et al. 2014), characterized by gradients in sea surface temperature and salinity, leading to gradual species presence adapted to varying conditions (Barahona et al. 2019; Hooker 2009; Hooker et al. 2014). This transition zone allows contact between closely related populations or species and may facilitate admixture through the mixing of genetically differentiated lineages, as has been observed in other marine contact zones (Helmerson et al. 2023; Jossey et al. 2024; Salas et al. 2020; Zbawicka et al. 2022). This secondary contact may be facilitated by ‘El Niño’–Southern Oscillation (ENSO) events, which weaken and displace the boundary between both currents by altering the sea surface temperature in the HCLME, allowing species to reach southern latitudes and thus fostering connectivity even beyond the transition zone (Arntz et al. 2006; Hooker 2009; Ñiquen and Bouchon 2004).

The Eastern Pacific harbors two closely related octopus species, *Octopus mimus* A. Gould, 1852, which inhabits the cold waters of the HCLME (Guerra et al. 1999; Norman et al. 2014), and *Octopus hubbsorum* S. S. Berry, 1953, which inhabits the warmer waters of the northeastern and central Pacific (Domínguez‐Contreras et al. 2013; Dueñas‐Romero et al. 2021). Genetic studies have revealed low genetic divergence between these species, leading some researchers to suggest they may represent a single species, *O. mimus* , with population‐level genetic structure (Ortiz‐Alvarez et al. 2026; Pardo‐Gandarillas et al. 2018; Pliego‐Cárdenas et al. 2020; Valdez‐Cibrián et al. 2020). These species could be considered as a species complex associated with varied environmental conditions, with *O. hubbsorum* associated with the Panamic province (from the Gulf of California, Mexico to the SEC) and *O. mimus* associated with the Peruvian province (from Peru to Chile) (Pliego‐Cárdenas et al. 2016). Both species are potentially in contact in the transition zone between the SEC and HCLME, in Peru.

Based on the convergence of the HCLME and SEC off the northern coast of Peru, we hypothesize that this environment promotes admixture between the genetically distinct species *O. mimus* and *O. hubbsorum* . For that, we generated and analyzed whole‐genome sequencing (WGS) data from 57 individuals along the coast of Peru, spanning from SEC to HCLME passing through the transition zone. Additionally, we broadened our latitudinal perspective to include the northernmost range of *O. hubbsorum*, incorporating 10 *O. hubbsorum* samples from Mexico, where the species was first described. This study aims to enhance our understanding of the evolutionary biology processes occurring in this transition zone, in the context of environmental variability within the Southeast Pacific region.

## 2 Materials and Methods

### 2.1 Sample Collection

A total of 67 tissue samples from *O. mimus–O. hubbsorum* complex individuals were collected at different locations. In Peru, sampling was conducted across three main regions. The north Peru (SEC) samples include those from Zorritos (*n* = 2), Punta Sal (*n* = 4), Los Órganos (*n* = 5), and El Ñuro (*n* = 6). The transition zone samples comprise those from Sechura (*n* = 4), Islas Lobos de Tierra (*n* = 4), and Islas Lobos de Afuera (*n* = 14). In HCLME, samples were collected from Huarmey (*n* = 4), Callao (*n* = 3), Ica (*n* = 3), Matarini (Arequipa) (*n* = 4), and Ilo (*n* = 4) (Figure 1; Table S1). In Mexico, samples were obtained from San Bruno (*n* = 10). Samples were preserved in 96% ethanol and stored at −20°C at the Universidad Nacional Mayor de San Marcos.

### 2.2 DNA Extraction

Genomic DNA was extracted from 5 mm^3^ of arm tissue using the Wizard SV Promega kit, following the manufacturer's protocol. DNA yield was quantified using a NanoDrop Lite spectrophotometer. DNA integrity was assessed through electrophoresis on a 1% agarose gel.

### 2.3 Whole‐Genome Sequencing Library Preparation and Sequencing

WGS libraries from 2018 to 2020 samples were prepared at the Norwegian University of Science and Technology (NTNU) using the BEST (blunt‐end single tube) library build protocol (Carøe et al. 2018). DNA input to the library build was approximately 500 ng when available. End‐repair, adapter ligation, and library amplification were performed following the single‐tube workflow described in the BEST protocol. Indexing PCR was carried out using dual‐index primers and AmpliTaq Gold DNA Polymerase (Applied Biosystems), with an initial activation step at 95°C for 10 min, followed by 12–14 cycles of 95°C for 30 s, 60°C for 30 s, and 72°C for 30 s, and a final extension at 72°C for 5 min. Amplified libraries were purified with Cytiva Sera‐Mag magnetic beads at a 1.67:1 bead‐to‐library ratio. Indexed libraries were quantified on an Agilent 4200 TapeStation instrument, pooled in equimolar concentrations, and sequenced by Novogene Europe on the NovaSeq X platform, generating 150‐bp read pairs. Library preparation, QC control, and WGS (NovaSeq X, PE150) from remaining samples were obtained via commercial service at Novogene USA.

### 2.4 Mapping and Reference Alignment

Raw sequencing reads were processed using the paleomix bioinformatics pipeline (Schubert et al. 2014). Sequence data was quality‐checked and processed using AdapterRemoval v2 (Schubert et al. 2016) to remove residual adapter sequences and low‐quality bases, as well as to merge overlapping read pairs into a single sequence when a minimum overlap of 11 bp was detected and to recalculate quality scores (option *‐‐collapse*). Cleaned reads were mapped to the *Octopus sinensis* reference nuclear genome (GCF_006345805.1), a relatively phylogenetically close species (Amor et al. 2017) with a high‐quality chromosome‐level assembly, using BWA v0.7.16 (mem algorithm) with default parameters (Li 2013). Only reads with high‐quality mappings (MAPQ score ≥ 30) were retained. PCR duplicates were removed using picard tools v2.21.2 (Scholak et al. 2021).

### 2.5 Mitogenome Assembly and Phylogenetic Analysis

To evaluate nuclear‐mitochondrial discordance, we reconstructed a mitochondrial‐based phylogeny. Subsequently, the mitochondrial genomes were assembled from the adapter‐trimmed reads using NovoPlasty v4.2 (Dierckxsens et al. 2016). The MITOS2 Web Server (Bernt et al. 2013) was employed for automated mitochondrial genome annotation. Protein‐coding genes were manually corrected using the publicly available reference mitochondrial genome of *O. hubbsorum* (NC_044093 from Mexico). We aligned our 67 mitochondrial sequences, the sequence of *O. hubbsorum* (NC_044093), and *O. sinensis* (NC_052881) as an outgroup using Muscle v3 (Edgar 2004). A phylogeny was inferred using IQ‐Tree v2.0.3 (Minh et al. 2020) with 1000 ultrafast bootstrap replicates and automatic determination of the best partition scheme and substitution models using ModelFinder based on the Bayesian Information Criterion (BIC).

### 2.6 SNP Discovery, Genotype Likelihood Estimation, and Heterozygosity Estimation

SNP discovery was conducted using ANGSD v0.935 (Korneliussen et al. 2014), with input files comprising BAM files derived from the aligned sequencing reads. A minimum mapping quality (MQ) of 30 was required, and only uniquely mapped reads were taken into consideration (read‐level filters). When considering variants, a minimum base quality (Q) of 20 filter was applied, and a minimum allele frequency of 0.05 was required for downstream analyses. SNPs were further subjected to quality filtering using a *p*‐value threshold of 1e‐6 (site‐level filters). Genome‐wide heterozygosity was estimated independently for each individual using ANGSD v0.935. Genotype likelihoods were computed for each BAM file using the Samtools model, and a folded site frequency spectrum (SFS) was generated with realSFS (−fold 1). Then, individual heterozygosity was calculated as the proportion of heterozygous sites inferred from the folded SFS. The *Octopus sinensis* genome (GCF_006345805.1) was used as both the reference and the ancestral sequence. Differences in genome‐wide heterozygosity among the North, South, Admixed‐Cold, and Admixed‐Warm genetic groups were assessed using pairwise Wilcoxon rank‐sum tests. Statistical comparisons and graphical annotations were implemented in R using the stat_compare_means function from the ggpubr package (Kassambara 2018).

The average sequencing depth was low (~1.01× per individual). However, genotype likelihood‐based approaches implemented in ANGSD are designed for low‐coverage population genomic data. These approaches have been used in population structure, admixture, and selection analyses, eliminating the need for explicit genotype calling (Korneliussen et al. 2014). Rather than relying on potentially error‐prone hard genotype calls, ANGSD incorporates genotype uncertainty directly into downstream analyses, thereby reducing biases associated with low sequencing depth. To minimize potential artifacts further, stringent filtering criteria were applied, including minimum mapping and base qualities, SNP significance thresholds, and minor allele frequency filters. Additionally, analyses were restricted to sites represented across a large proportion of individuals, thereby reducing the influence of missing data and low‐confidence variants. Similar low‐coverage genomic approaches have been successfully applied in previous evolutionary and population genomic studies using marine and non‐model organisms (Bieker et al. 2022; Lou et al. 2021; Warmuth and Ellegren 2019).

### 2.7 Population Structure Analysis

The admixture analysis was conducted using the software NGSadmix v.1.0 (Skotte et al. 2013), testing *K* values from 2 to 10. The analyses were conducted on a BEAGLE‐formatted genotype file where sites on the mitochondrial DNA contigs were filtered out. The minimum allele frequency was set at 0.1, and the minimum sample size was set to 34 individuals. The plots were generated using the R package ggplot2. We estimated the most probable number of clusters (*K*) using the Evanno method (Earl and vonHoldt 2012).

To investigate the genetic structure of the *O. mimus* – *O. hubbsorum* complex, a Principal Component Analysis (PCA) was performed using PCAngsd (Meisner and Albrechtsen 2018) which estimates covariance matrices directly from genotype likelihoods. Prior to PCA, single nucleotide polymorphisms (SNPs) were pruned for linkage disequilibrium (LD) using PLINK v1.9 (Chang et al. 2015) with the indep‐pairwise algorithm (window size = 50 SNPs, step size = 5 SNPs, *r* ^2^ threshold = 0.5). PCAngsd was run for 10,000 iterations using 40 threads, and the resulting covariance matrix was used to compute principal components. PCA results were visualized in R using the ggplot2 package, plotting the first two principal components and coloring individuals according to the population clusters inferred from the NGSadmix analysis for *K* = 4.

### 2.8 Admixture Analyses

To test the hypothesis that the Transition Zone population of octopus represents an admixture of *O. hubbsorum* and *O. mimus* populations, we used F3 statistics implemented in Admixtools package (Patterson et al. 2012). For this analysis, we clustered the individuals into four distinct populations as follows: *O. hubbsorum* , Admixed‐Cold, Admixed‐Warm, and *O. mimus* (Table S1). We excluded individuals CCM014, APN48, and VOE12 from the F3 analyses because, relative to the remaining admixed individuals, their ancestry profiles suggested more complex multigenerational admixture patterns. These individuals showed intermediate genomic compositions that differed from the main Admixed‐Cold and Admixed‐Warm clusters in both the PCA and NGSadmix analyses, potentially reflecting secondary admixture among already admixed lineages. Since F3 statistics are designed to test admixture scenarios that assume relatively well‐defined source and recipient populations, including these individuals could bias the inference of admixture signals and reduce the interpretability of population‐level comparisons. Thus, these individuals were excluded from only the F3 analyses while remaining included in the broader population genomic analyses. Furthermore, the Z‐score for each F3 statistic was computed to ascertain the significance of the observed values. Negative F3 values suggest potential admixture or historical gene flow between populations, indicating that a population could be a mixture of two ancestral groups.

In order to detect recent admixture (i.e., within the last five generations), we used the apoh (admixture pedigrees of hybrids) approach (Garcia‐Erill et al. 2023). This software uses estimates of paired ancestry proportions to detect recently admixed individuals and possible scenarios of admixture pedigrees. This approach has been demonstrated to be reliable in data sets with reduced resource availability, such as low‐depth sequencing data (Garcia‐Erill et al. 2023). Paired ancestry estimates were calculated using the allele frequencies calculated in the previous NGSadmix (*K* = 2) analysis, enabling the estimation of both parental ancestries and the full paired‐ancestry matrix for each individual.

### 2.9 Outlier Analyses

To investigate whether adaptive divergence contributes to the genomic differentiation observed between *O. mimus and O. hubbsorum*, we performed selection signature scans to identify SNPs showing unusually high differentiation between the two species. These analyses were intended to detect signatures of divergent selection associated with contrasting environmental conditions between the South Equatorial Current (SEC) and the Humboldt Current Large Marine Ecosystem (HCLME), rather than to infer additional population structure.

We performed selection signature scans between species using PCAngsd v1.10 with the ‐selection option (Meisner et al. 2021), which extends FastPCA (Galinsky et al. 2016) for use with low‐depth sequencing data. Only pure individuals identified by NGSadmix and APOH analyses were included in the comparison using the LD‐filtering SNP dataset employed for NGSadmix analyses. The comparison was performed between the parental clusters assigned to *O. hubbsorum and O. mimus*. Two eigenvectors were specified, and outlier loci were identified using chi‐square tests (df = 1), with *p*‐values adjusted using Bonferroni correction (α = 0.05). A dataset containing only the identified outlier SNPs was generated for all individuals and used for additional PCA and NGSadmix analyses to evaluate differentiation patterns across admixed and parental groups.

## 3 Results

Following quality filtering and alignment to the *O. sinensis* reference genome, sequencing depth across individuals averaged 1.01×, after excluding reads with mapping quality below 30. Prior to linkage disequilibrium (LD) pruning, 198,358 sites were retained. After LD pruning, 151,782 sites remained, and subsequent removal of mitochondrial loci resulted in a final dataset of 151,779 biallelic SNPs. This dataset was used for population genomic analyses, including PCA, NGSadmix, and outlier detection.

**FIGURE 2:**
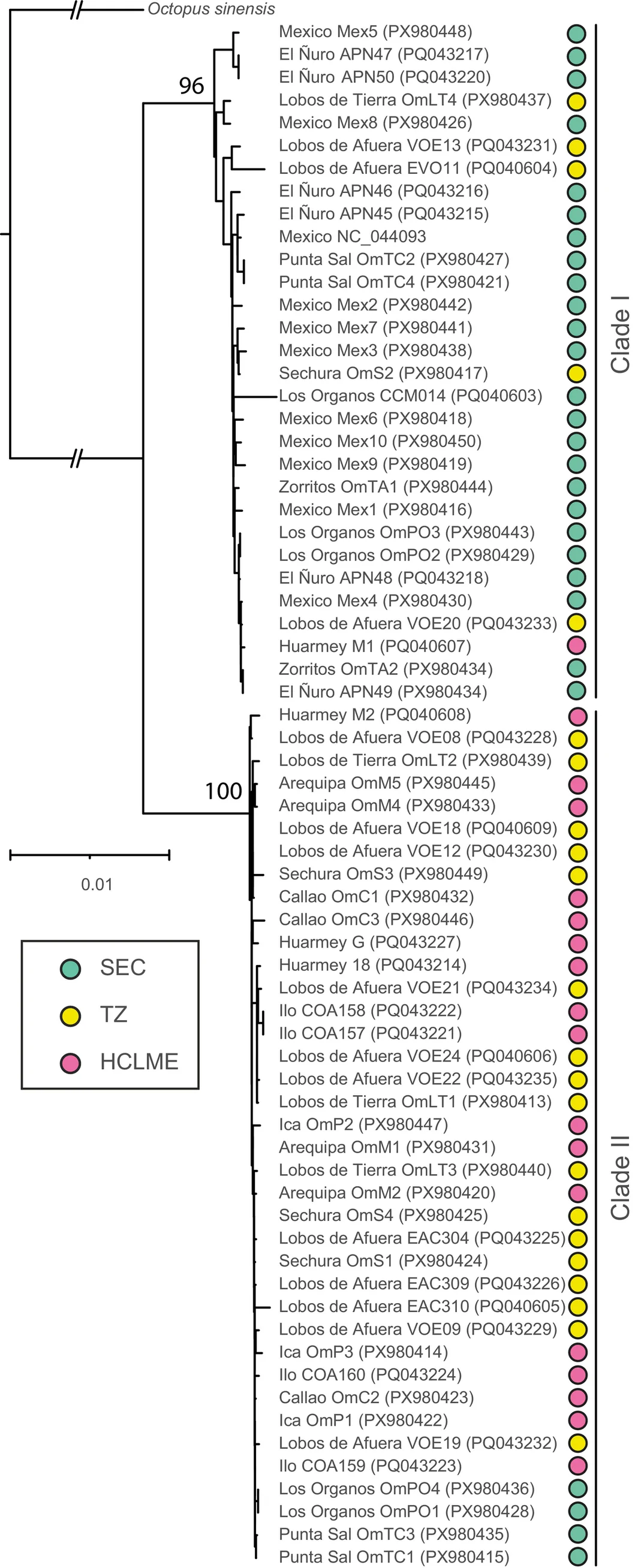
Phylogenetic tree of *O. mimus* — *O. hubbsorum* complex based on mitochondrial genome sequences. The tree is rooted with *O. sinensis* as the outgroup. The scale bar represents 0.01 substitutions per site. South Equatorial Current (SEC), Transition Zone (TZ), and the Humboldt Current Large Marine Ecosystem (HCLME).

We obtained 60 fully circularized mitochondrial genomes and 7 incomplete mitochondrial genomes (> 9000 bp). Phylogenetic analysis of the aligned mitochondrial genome sequences identified two major clades (Figure 2). Most individuals from the SEC and Mexico populations were classified in Clade I, except four SEC samples. Specimens from the HCLME population were mainly in Clade II, excluding sample M1. Transition zone samples are found in both Clade I and Clade II (Figure 2).

**FIGURE 3:**
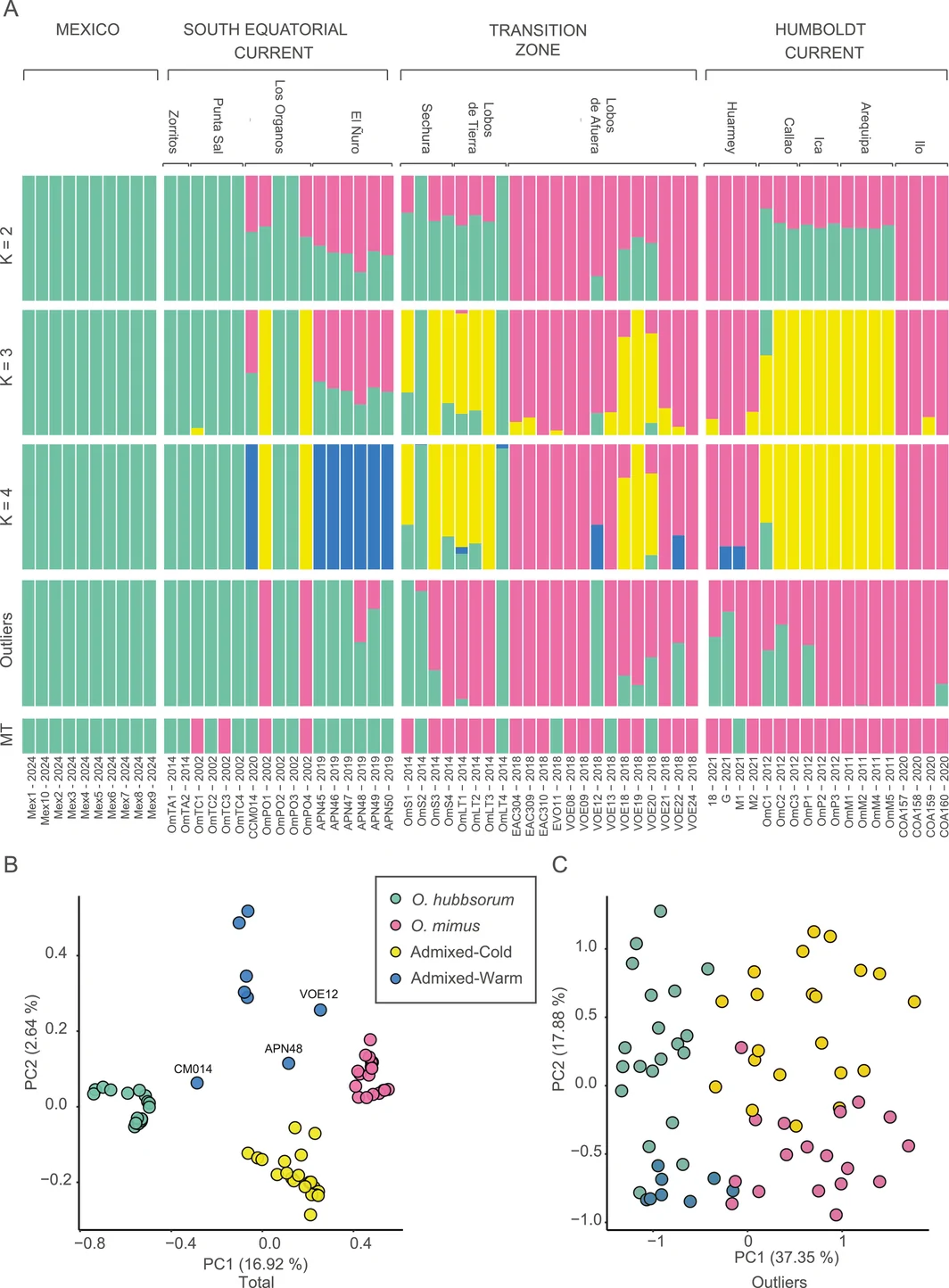
Genetic structure between *O. mimus–O. hubbsorum* populations along the Peruvian coast. (A) NGSadmix using whole genome sequencing WGS data for *K* = 2, *K* = 3, *K* = 4, and *K* = 2 only using outliers SNPs from PCangsd. MT represents the mitochondrial clades. The bars represent individual genetic assignments. (B) PCA analysis using all sites after filtering. (C) PCA analysis using only outlier SNPs from the selection scan.

Genome‐wide structure analysis indicated that the most likely number of genetic clusters was two (*K* = 2) (Figure S1 and Figure 3), *O. hubbsorum* (Mexico and SEC) and *O. mimus* (HCLME). An admixture zone was identified between the North and South populations, where individuals showed different degrees of genetic identity to both groups. Mexican individuals, as well as those from the northernmost sites of the Peruvian coast (Zorritos and Punta Sal) and the southernmost site (Ilo), showed pure genetic composition. Notably, both pure and admixed specimens were identified among the SEC, HCLME, and transition zone populations. At *K* = 3 and *K* = 4, the presence of pure individuals at the geographic extremes remained well defined, while admixed individuals constituted new genetic clusters here named Admixed‐Cold and Admixed‐Warm (Figure 3A).

The distribution of samples in PCA space was highly consistent with the admixture results, reinforcing the existence of two primary genetic clusters with a gradient of admixture between them (Figure 3B). North and South populations formed well‐defined clusters along PC1, which accounted for 16.92% of the total genetic variance. Individuals categorized as Admixed‐Cold and Admixed‐Warm were positioned in an intermediate position between these two groups (equidistance on PC1), being differentiated along PC2 (2.64% of the variance).

**FIGURE 4:**
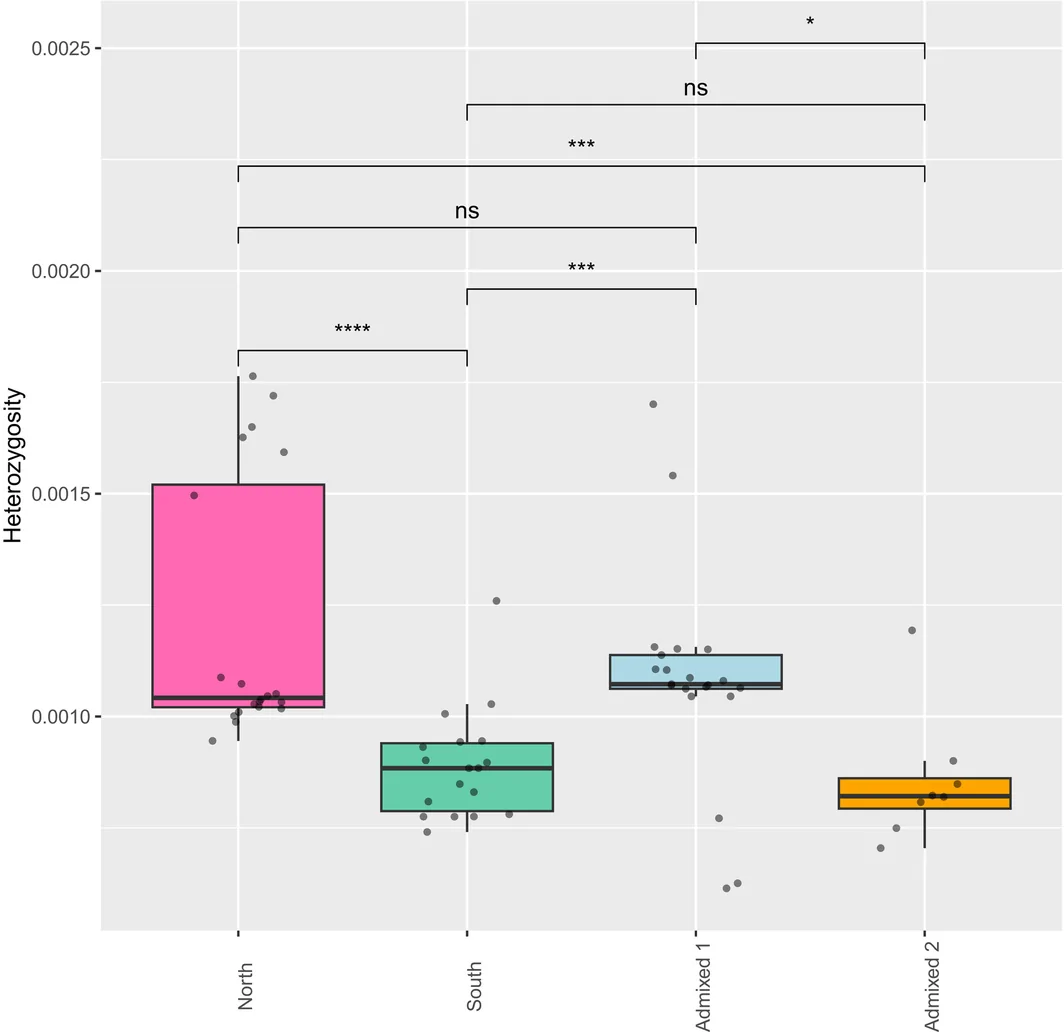
Genome‐wide heterozygosity across genetic clusters of *O. mimus* and *O. hubbsorum* populations. Boxplots show heterozygosity values for each group. A Wilcoxon rank sum test was used to test for significant differences between populations. Significance levels are indicated as: *p* < 0.05 (*), *p* < 0.001 (***), *p* < 0.0001 (****), and ns = not significant.

Genome‐wide heterozygosity (*H*_*e*_) estimates exhibited variability across different genetic clusters (Figure 4). The highest mean heterozygosity was observed in *O. hubbsorum* (Mexico and SEC) and *O. mimus* , with no significant differences between the two species. Whereas both admixed clusters (Admixed–Cold and Admixed–Warm) showed significantly lower heterozygosity values when compared with the pure populations. No significant differences were detected between the two admixed clusters (Figure 4).

The f3 results suggest that the Admixed‐Cold and Admixed‐Warm populations could be interpreted as a genetic admixture between *O. hubbsorum* and *O. mimus* . In this context, these populations are the recipient populations, while the North and South populations are potential source populations (Table 1).

**TABLE 1:** F3 statistics for population comparisons in *O. mimus–O. hubbsorum* com*plex*.

Pop 1	Pop 2	Pop 3	F3	*z*	*p*
Admixed‐Cold	Admixed‐Warm	*O. hubbsorum*	0.0008	4.7949	< 0.00001
Admixed‐Cold	Admixed‐Warm	*O. mimus*	0.0001	0.94	0.3472
**Admixed‐Cold**	*O. hubbsorum*	*O. mimus*	**−0.0017**	**−13.2723**	**< 0.00001**
Admixed‐Warm	*O. hubbsorum*	Admixed‐Cold	0.0015	16.6076	< 0.00001
**Admixed‐Warm**	*O. hubbsorum*	*O. mimus*	**−0.0004**	**−8.8614**	**< 0.00001**
Admixed‐Warm	*O. mimus*	Admixed‐Cold	0.0022	15.3297	< 0.00001

*Note:* Admixed populations are indicated by negative values with |*z*| > 3 (bold).

**FIGURE 5:**
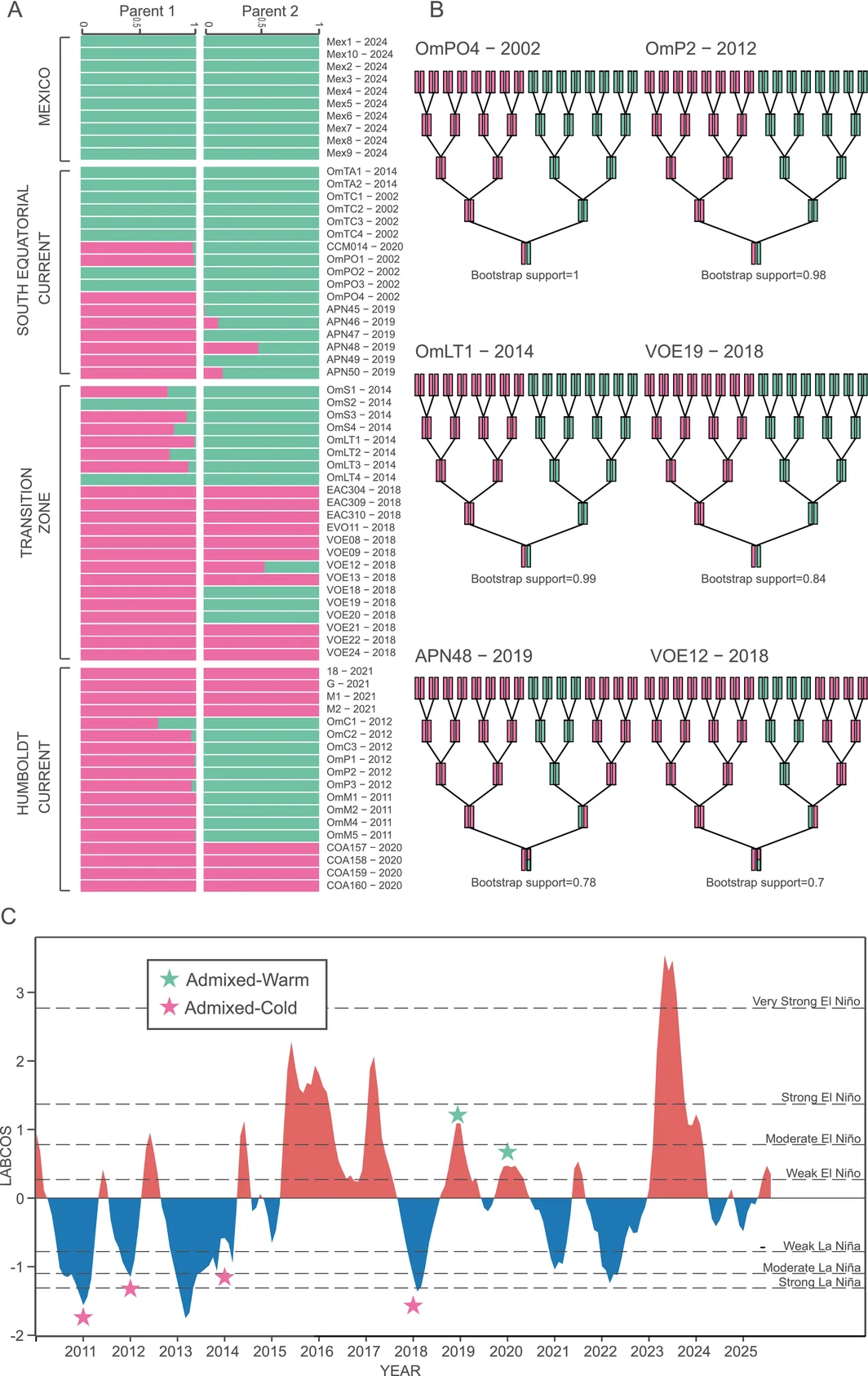
Admixture dynamics and ENSO‐associated climatic variability in the *Octopus mimus–O. hubbsorum* complex. (A) Estimates for recent admixture. Admixture proportion of parental genetic clusters, based on *K* = 2, using apoh. Individual identifiers are indicated beneath each bar. (B) Most compatible admixture pedigrees, based on bootstrapping, showing F1 and F2 individuals sampled in different years. The individual's identifier is indicated above its pedigree. (C) Índice Laboratorios Costeros (LABCOS) showing variability on superficial temperature in the coastal zone (Quispe Sánchez and Vásquez Espinoza 2015). The stars indicate the years where admixed individuals were sampled.

Recent admixture events were detected in all individuals from Admixed‐Cold or Admixed‐Warm. Most of these individuals were suggested as F1 (admixture index = 1), while two individuals (APN48 and VOE12) have admixture index values close to 2 (F2). In contrast, individuals of *O. hubbsorum* and pure *O. mimus* populations show no evidence of recent admixture (Figure 5A,B).

The selection scan revealed 67 outlier SNPs that underlie differences between *O. hubbsorum* and *O. mimus* populations. Structure analysis supported *K* = 2 populations (Figure 3A). Considering the candidate outlier SNP dataset, Admixed‐Cold individuals show predominantly *O. mimus* ancestry, while Admixed‐Warm shows *O. hubbsorum* ancestry. Similarly, PCA shows the same pattern in PC1 (Figure 3C).

## 4 Discussion

Research on admixture dynamics in marine contact zones has attracted significant attention within the scientific community due to its pivotal role in shaping the genetic landscape of marine ecosystems, providing valuable insights into the evolutionary processes governing species diversification and adaptation (Faria et al. 2021; Moritz et al. 2009; Pérez‐Portela et al. 2017). Our results support the genetic structure maintained by ocean currents in the *O. mimus–O. hubbsorum* complex, in both the mitochondrial and nuclear genomes. This mitochondrial genome structure has been previously demonstrated using the genes COI, COIII, and ND5, revealing a low level of genetic divergence between populations (Domínguez‐Contreras et al. 2018; Dueñas‐Romero et al. 2021; Pardo‐Gandarillas et al. 2018; Pliego‐Cárdenas et al. 2014, 2016, 2020). The presence of distinct genetic clusters along the latitudinal gradient supports the existence of two genetic groups, one likely adapted to warmer northern waters corresponding to the South Equatorial Current (SEC) and another to colder southern waters associated with the Humboldt Current System (HCLME). However, in the transition zone between these currents, the presence of admixed individuals was observed (Figure 3). Similar patterns of admixture and genetic exchange, akin to those observed in octopus populations, have been documented in various marine organisms such as mussels, whales, and anchovies, particularly in areas where different populations converge (Fraïsse et al. 2021; Hudson et al. 2020; Le Moan et al. 2016; Violi et al. 2023), such as transition zones between ocean currents or regions characterized by complex bathymetry (Barth et al. 2017).

The admixture identified in this study seems to be dynamic, exhibiting two distinct genetic admixed clusters, here referred to as Admixed‐Cold and Admixed‐Warm (Figure 2). Interestingly, both admixed clusters appear to derive from admixture between *O. hubbsorum* and *O. mimus* populations (Table 1); however, observed genetic differences indicate a more complex scenario. This complexity is further illustrated in the PCA analysis, where a “V” pattern, indicative of hybrid origin, is apparent for each cluster (Elgvin et al. 2017; Lopes et al. 2023). The two admixed clusters occupy intermediate positions between parental populations along PC1 and show differences in genetic composition on PC2. This variation may reflect segregation of alternative alleles inherited from the parental populations and/or divergence associated with selective or demographic processes (Elgvin et al. 2017). Our findings suggest that part of the genetic differentiation between the admixed clusters may reflect divergent environmental or selective processes. The NGSadmix analysis based on outlier SNPs supports this interpretation. Admixed‐Cold individuals clustered more closely with *O. mimus*, and Admixed‐Warm individuals showed greater affinity with *O. hubbsorum* (Figure 3A).

The admixture pattern observed in this study may be associated with large‐scale climatic variability, such as ENSO events. These events can alter oceanographic boundaries, potentially facilitating larval dispersal and connectivity between regions and contributing to genetic exchange and hybridization (Pardo‐Gandarillas et al. 2018). *Octopus* produces a high number of small eggs that hatch into planktonic paralarvae (Alejo‐Plata and Alejo 2014). In contrast to the adults, which display sedentary behavior, these paralarvae possess the capacity for extensive dispersal. Their dispersal occurs during the planktonic phase and is closely associated with ocean currents (Villanueva and Norman 2008). Under typical circumstances, the upwelling of cold waters in the Humboldt Current impedes the southward movement of northern individuals, representing a natural barrier. However, during El Niño events, the weakening of upwelling systems and the intrusion of warmer waters temporarily relax this barrier, allowing for increased connectivity (Glantz 2001). Thus, paralarvae from the northern populations ( *O. hubbsorum* ) may disperse into the Humboldt current and settle to mature as sedentary adults, achieving sexual maturity after a brief period of benthic growth lasting six to twelve months (Olivares et al. 1996). This process can lead to the production of hybrid paralarvae with local *O. mimus* populations in the subsequent generation. In contrast, La Niña events cause the cold water to shift westward, leading to lower than usual sea surface temperatures and altering the barrier, which facilitates the dispersal of paralarvae from southern populations ( *O. mimus* ) to areas north of the transition zone, which could lead to the formation of hybrids. Thus, ENSO events could influence the admixture processes during both the warming phase (El Niño) and the cooling phase (La Niña).

This hybridization phenomenon could have significant implications for adaptation and fitness, given that each parental population is adapted to specific oceanographic conditions. Hybrids formed through gene mixing may inherit a combination of traits, which could prove disadvantageous if adaptive characteristics from parent populations become diluted (Rudman and Schluter 2016). Since ENSO events typically last less than one year (Takahashi and Dewitte 2016) and environmental conditions revert to normal, only hybrid individuals possessing sufficient adaptive traits after allele segregation are likely to survive to adulthood. This could be observed in Figure 3C, where all the Admixed‐Cold individuals share most of the adaptive genetic diversity of *O. mimus* (PC1) compared to *O. hubbsorum* (PC2), while the Admixed‐Warm loads mainly the *O. hubbsorum* adaptive component. Therefore, the survival of admixed individuals formed during ENSO events is likely determined by the adaptive genetic composition of each organism and the prevailing environmental conditions, particularly during the early stages of life.

This hypothesis of purifying selection is supported by several observations: (1) The observation that all Admixed‐Cold individuals were sampled under years with cold conditions (La Niña), whereas Admixed‐Warm individuals were found during El Niño years (warm environment) (Figure 5C). (2) The outliers SNPs found have been previously related to biological pathways involved in environmental stress responses (Table S2), including genes involved in thermal stress and oxidative regulation in marine organisms subjected to high temperatures (Rosic et al. 2010), cellular stress responses under heat stress conditions in marine algae (Wang et al. 2022), and epigenetic regulation and heat shock responses in marine invertebrates (Xu and Wang 2019). This result supports the hypothesis that some differentiated loci may be associated with biological processes involved in environmental adaptation under the fluctuating oceanographic conditions characteristic of the ENSO‐influenced transition zone. (3) The loss of heterozygosity in the admixed populations (Figure 4), as expected in the case of purifying selection. In specific, some hybrid genomic combinations are differentially selected across environmental conditions, with maladaptive combinations being purged, resulting in reduced heterozygosity and potentially producing individuals that are genetically pure in the nuclear genome but possess a divergent mitochondrial lineage (e.g., OmTC3 and EVO11 in Figure 3). (4) The admixture pedigree analysis identified most individuals as F1, only two F2 individuals, but no F3 to F5 individuals, which may suggest differential persistence among hybrid genomic backgrounds or even hybrid breakdown. Notably, the F1 individuals were sampled over multiple years between 2002 and 2020, suggesting that admixture events may occur repeatedly through time under fluctuating oceanographic conditions.

The admixture pattern observed herein is not necessarily exclusive to *O. mimus* – *O. hubbsorum* complex, as this area serves as the habitat of many marine species. Considering these observations, in conjunction with the ENSO events, the transition zone can be viewed as acting as a natural evolutionary laboratory where different species and populations interact and evolve. Given the challenges posed by environmental changes and human impacts, it is imperative to prioritize conservation efforts to safeguard such transitional habitats. Currently, management measures in Peru for the *O. mimus* – *O. hubbsorum* complex are largely uniform throughout its range, establishing quotas and seasonal bans without accounting for potential genetically distinct populations. However, in some marine protected areas (MPAs) where octopus fisheries occur (e.g., Ilo), management measures include locally defined quotas and fishing seasons that are updated annually (Ministerial Resolution No. 512‐2024‐PRODUCE). These findings indicate that fishery management could be more adaptive by employing genetic information to identify populations requiring specific conservation measures. This approach could enhance the sustainable use of the resource and the long‐term viability of this important species (Del Solar et al. 2023). Notably, the recent establishment of the National Marine Reserve Mar Tropical de Grau by the Peruvian Government, through Supreme Decree No. 003‐2024‐MINAM, represents a significant advancement in marine biodiversity conservation and sustainable resource management, particularly within the tropical‐temperate transition zone. Although El Ñuro is currently the only sampling site under protection, Isla Foca, located within the transition zone (Cutipa‐Luque et al. 2020) has also been included within the boundaries of the newly established reserve, thereby demonstrating the government's commitment to expanding conservation efforts. The integration of genetic data with ecological and oceanographic information presents an opportunity to deepen our understanding of the processes influencing genetic diversity and population connectivity in these contact zones.

Some limitations of this study should be acknowledged, along with recommendations for future research. First, because samples were collected over two decades, temporal variation may have influenced the inferred genetic structure, potentially reflecting differences among sampling years rather than purely spatial population structure. However, the genetic clusters were detected across multiple years, suggesting that the observed structure is not mainly driven by temporal sampling effects. In addition, the sampling design was not intended to test admixture dynamics across ENSO phases but rather resulted from individual research efforts aimed at future taxonomic studies. Nevertheless, pure and admixed individuals were sampled in the same years during ENSO periods (e.g., 2002, 2014, 2018, and 2020). Future studies with broader geographic coverage and systematic temporal sampling across contrasting ENSO phases will be necessary to infer and quantify the frequency and extent of hybridization events and their relationship with ENSO phases. Finally, the proposed hypothesis of admixture and purifying selection during ENSO events is supported by correlations between ENSO phases and the occurrence of admixed individuals, thermal‐stress‐related outlier SNPs, reduced heterozygosity in admixed individuals, and the recurrent detection of F1 individuals during ENSO years. However, future studies should incorporate direct fitness or survival data to explicitly test and confirm the purifying selection hypothesis presented here.

In conclusion, our results support the hypothesis that the transition zone between North and South influences the genetic structure of *O. mimus* – *O. hubbsorum* complex. The presence of two clades in our nuclear and mitochondrial data and the observed admixture in our nuclear data suggests an intricate evolutionary scenario in *O. mimus* – *O. hubbsorum* complex and provides evidence that the genetic differences between the North and South populations have likely been maintained over time through a combination of oceanographic barriers, episodic connectivity, and selective processes. Our results suggest that admixture in the transition zone may represent a dynamic process influenced by environmental variability and episodic connectivity. The observed genomic patterns are consistent with the hypothesis that climatic fluctuations such as ENSO‐associated oceanographic variability may contribute to episodic connectivity and admixture in the transition zone. These findings contribute to a growing body of evidence supporting the idea that marine transition zones function as natural evolutionary laboratories, where selection, gene flow, and environmental forces interact to shape biodiversity. Finally, considering *O. mimus* and *O. hubbsorum* as a species complex, this finding has important implications for understanding ongoing speciation dynamics. In that sense, *O. mimus* and *O. hubbsorum* could be considered as two different species adapted to different environmental conditions, that interact in the contact zone, forming hybrid individuals that may experience variable evolutionary outcomes depending on environmental and genomic context, being currently in a gray zone of speciation. Future genomic and ecological studies incorporating temporal sampling across different ENSO phases will be essential to fully understand the cyclical nature of admixture, selection, and population connectivity in this transition zone.

## Author Contributions

**Jackeline Ruiz‐Mendoza:** conceptualization (equal), formal analysis (equal), writing – original draft (equal), writing – review and editing (equal). **Vanessa C. Bieker:** formal analysis (equal), supervision (equal), writing – review and editing (equal). **Jaime G. Morin‐Lagos:** formal analysis (equal), writing – review and editing (equal). **Franz Cardoso:** writing – review and editing (equal). **Ximena Vélez‐Zuazo:** conceptualization (equal), writing – review and editing (equal). **Joanna Alfaro‐Shigueto:** conceptualization (equal), funding acquisition (equal), writing – review and editing (equal). **Marcial Arellano‐Martínez:** writing – review and editing (equal). **Raquel Siccha‐Ramirez:** conceptualization (equal), funding acquisition (equal), writing – review and editing (equal). **Michael D. Martin:** conceptualization (equal), formal analysis (equal), funding acquisition (equal), supervision (equal), writing – review and editing (equal). **Jorge L. Ramirez:** conceptualization (equal), formal analysis (equal), funding acquisition (equal), supervision (equal), writing – original draft (equal), writing – review and editing (equal).

## Funding

This study was funded by the Fondo Nacional de Desarrollo Científico, Tecnológico y de Innovación Tecnológica, Consejo Nacional de Ciencia, Tecnología e Innovación Tecnológica (CONCYTEC) and the Programa Nacional de Investigación Científica y Estudios Avanzados (PROCIENCIA) within the framework of the “Proyectos de Investigación Básica 2023‐01” contest, grant number PE501082404‐2023. Additional funding was provided by NORPART award 2021/10475 from the Norwegian Directorate for Higher Education and Skills via the ‘BiGTREE’ project.

## Ethics Statement

All procedures conducted in this study were carried out in strict accordance with the regulations outlined in the permit granted by the Dirección General de Autorizaciones en Acuicultura y Manejo de Peces Ornamentales (DGAAAMPA) of the Ministerio de la Producción del Perú No. 00077503‐2020‐E. The ethical principles and guidelines established by DGAAAMPA were strictly adhered to throughout the study.

## Conflicts of Interest

The authors declare no conflicts of interest.

## Supporting information


**Table S1:** Sample database with their respective location and sampling date. North, south (HCLME), transition zone (TZ).

**Table S2:** Annotated product of candidate loci under selection and their potential association with environmental stress responses.

**Figure S1:** The Evanno method output (ΔK) for NGSadmix runs.

## Data Availability

The raw sequence reads obtained in this study have been deposited in the NCBI Sequence Read Archive (SRA) under BioProject accession PRJNA1110282. The mitogenome sequences have been deposited on NCBI GenBank (PQ040603‐09; PQ043214‐35; PX980413‐450).
