# Supplementary material for: Admixture and Selection Driven by El Niño–Southern Oscillation Events Shape the Genetic Structure of Octopus mimus–O. hubbsorum Complex Across the Humboldt and South Equatorial Current Transition Zone
Source: Ecol Evol. 2026 Sep 2;16(9):e74222. doi: 10.1002/ece3.74222 (PMC13539069; doi:10.1002/ece3.74222)
Supplement: Supplementary file 1 [file ECE3-16-e74222-s001.docx]

Table S2. Annotated product of candidate loci under selection and their potential association with environmental stress responses.

| SNP | start | end | gene name | product | Description | Reference |
| --- | --- | --- | --- | --- | --- | --- |
| NC_042999.1_97299516 | 97287947 | 97437436 | LOC115209094 | thyroid receptor-interacting protein 11, transcript variant X1 | Transcriptional co-regulator potentially involved in gene expression regulation. | (Eirin-Lopez & Putnam, 2019) |
| NC_042999.1_97431057 | 97287947 | 97437436 | LOC115209094 | thyroid receptor-interacting protein 11, transcript variant X1 | Transcriptional co-regulator potentially involved in gene expression regulation. | (Eirin-Lopez & Putnam, 2019) |
| NC_042999.1_115428421 | 115415869 | 115435930 | LOC115209100 | pentatricopeptide repeat-containing protein 2, mitochondrial, transcript variant X1 |  |  |
| NC_042999.1_96207133 | 95180330 | 96283661 | LOC115209155 | protein still life, isoform SIF type 1, transcript variant X2 |  |  |
| NC_042999.1_129181726 | 129013879 | 129242751 | LOC115209179 | activated CDC42 kinase 1, transcript variant X6 |  |  |
| NC_042999.1_124852965 | 124841554 | 124933785 | LOC115209188 | transmembrane protein 94, transcript variant X1 |  |  |
| NC_042999.1_113106556 | 113052612 | 113110550 | LOC115209215 | uncharacterized LOC115209215, transcript variant X3 |  |  |
| NC_042999.1_98346941 | 98345155 | 98375017 | LOC115209229 | serine/threonine-protein kinase RIO3 | Regulate intracellular signalling pathways activated during environmental stress responses. | (Pérez‐Portela et al., 2020; Wang et al., 2022) |
| NC_042999.1_89789212 | 89767223 | 89836607 | LOC115209300 | tetratricopeptide repeat protein 21B |  |  |
| NC_042999.1_157512728 | 157466865 | 157519229 | LOC115209306 | serine/threonine-protein kinase 3 | Protein phosphorylation is a major mechanism regulating cellular responses to environmental stress. | (Wang et al., 2022) |
| NC_042999.1_98317373 | 98314887 | 98319259 | LOC115209369 | gastrula zinc finger protein XlCGF57.1-like |  |  |
| NC_042999.1_75780193 | 75720654 | 75806916 | LOC115209486 | cytochrome P450 2U1-like | participate in oxidative metabolism, detoxification and cellular responses to thermal and oxidative stress in marine organisms. | (Pankov et al., 2021; Rosic et al., 2010) |
| NC_042999.1_86064286 | 86025664 | 86291034 | LOC115209669 | protein Wnt-5b-like | Wnt signalling pathways are involved in developmental plasticity and may contribute to responses to environmental variability. | (Eirin-Lopez & Putnam, 2019) |
| NC_042999.1_75198043 | 75191466 | 75204174 | LOC115209814 | sulfotransferase 1E1-like |  |  |
| NC_042999.1_131743102 | 131662109 | 131824389 | LOC115209825 | uncharacterized LOC115209825, transcript variant X3 |  |  |
| NC_042999.1_131743157 | 131662109 | 131824389 | LOC115209825 | uncharacterized LOC115209825, transcript variant X3 |  |  |
| NC_042999.1_131743169 | 131662109 | 131824389 | LOC115209825 | uncharacterized LOC115209825, transcript variant X3 |  |  |
| NC_042999.1_78138318 | 77927540 | 78187167 | LOC115209828 | serine/threonine-protein phosphatase 2A 56 kDa regulatory subunit epsilon isoform, transcript variant X2 | Involved in stress response pathways. | (Wang et al., 2022) |
| NC_042999.1_96395076 | 96371483 | 96473468 | LOC115209845 | con-Ins Im1, transcript variant X1 |  |  |
| NC_042999.1_114201336 | 114157102 | 114343185 | LOC115209863 | histone acetyltransferase type B catalytic subunit, transcript variant X2 | Regulates gene expression during heat stress and environmental acclimation through epigenetic mechanisms. | (Fellous et al., 2022; Xu & Wang, 2019) |
| NC_042999.1_126524378 | 126516432 | 126561579 | LOC115209864 | F-box/SPRY domain-containing protein 1-like |  |  |
| NC_042997.1_32278345 | 32146315 | 32484507 | LOC115210466 | uncharacterized LOC115210466 |  |  |
| NC_043001.1_42726924 | 42694879 | 42758519 | LOC115211682 | proprotein convertase subtilisin/kexin type 7 |  |  |
| NC_042997.1_143076407 | 143005014 | 143112471 | LOC115211685 | SUN domain-containing ossification factor, transcript variant X3 |  |  |
| NC_043001.1_43472124 | 43444862 | 43472586 | LOC115211858 | uncharacterized LOC115211858, transcript variant X2 |  |  |
| NC_043001.1_122351393 | 122346969 | 122375159 | LOC115212039 | cytochrome P450 4V2-like | Involved in oxidative metabolism and environmental stress responses | (Pankov et al., 2021; Rosic et al., 2010) |
| NC_043001.1_121609310 | 121552253 | 121612066 | LOC115212295 | nuclear pore complex protein Nup160 |  |  |
| NC_043001.1_121609501 | 121552253 | 121612066 | LOC115212295 | nuclear pore complex protein Nup160 |  |  |
| NC_043002.1_45570719 | 45537833 | 45609150 | LOC115212823 | uncharacterized LOC115212823, transcript variant X2 |  |  |
| NC_043003.1_92774222 | 92728223 | 92809583 | LOC115213925 | kinesin-like protein KIF3B, transcript variant X4 |  |  |
| NC_043006.1_85774347 | 85766728 | 85851105 | LOC115216321 | F-actin-capping protein subunit beta |  |  |
| NC_042997.1_146877703 | 146870011 | 146896379 | LOC115217853 | cytochrome P450 20A1 | Frequently associated with physiological acclimation to environmental stressors. | (Pankov et al., 2021; Rosic et al., 2010) |
| NC_043010.1_61779558 | 61743746 | 61783773 | LOC115218854 | zinc finger protein 585A-like, transcript variant X2 | Regulate transcriptional responses to environmental stress. | (Eirin-Lopez & Putnam, 2019) |
| NC_043017.1_4380090 | 4272818 | 4470727 | LOC115222824 | protein vav, transcript variant X3 |  |  |
| NC_043020.1_29764426 | 29558329 | 29819091 | LOC115224129 | dihydropyrimidinase, transcript variant X9 |  |  |
| NC_043022.1_17869414 | 17813183 | 17935437 | LOC115224791 | rho guanine nucleotide exchange factor 26, transcript variant X3 |  |  |
| NC_042997.1_165809426 | 165787577 | 165844936 | LOC115228511 | coiled-coil domain-containing protein 146-like, transcript variant X5 |  |  |
| NW_021834759.1_13810 | 1352 | 28491 | LOC118761746 | uncharacterized LOC118761746 |  |  |
| NC_042997.1_21970583 |  |  | Intergenic region |  |  |  |
| NC_042998.1_142734969 |  |  | Intergenic region |  |  |  |
| NC_042998.1_149120553 |  |  | Intergenic region |  |  |  |
| NC_042999.1_101511640 |  |  | Intergenic region |  |  |  |
| NC_042999.1_109565720 |  |  | Intergenic region |  |  |  |
| NC_042999.1_110776133 |  |  | Intergenic region |  |  |  |
| NC_042999.1_113013677 |  |  | Intergenic region |  |  |  |
| NC_042999.1_113566735 |  |  | Intergenic region |  |  |  |
| NC_042999.1_154966330 |  |  | Intergenic region |  |  |  |
| NC_042999.1_35378557 |  |  | Intergenic region |  |  |  |
| NC_042999.1_68939852 |  |  | Intergenic region |  |  |  |
| NC_042999.1_70727630 |  |  | Intergenic region |  |  |  |
| NC_042999.1_70727702 |  |  | Intergenic region |  |  |  |
| NC_042999.1_70907999 |  |  | Intergenic region |  |  |  |
| NC_042999.1_78912618 |  |  | Intergenic region |  |  |  |
| NC_042999.1_87018326 |  |  | Intergenic region |  |  |  |
| NC_042999.1_96880976 |  |  | Intergenic region |  |  |  |
| NC_042999.1_96935537 |  |  | Intergenic region |  |  |  |
| NC_042999.1_96935587 |  |  | Intergenic region |  |  |  |
| NC_042999.1_97095044 |  |  | Intergenic region |  |  |  |
| NC_043001.1_118619076 |  |  | Intergenic region |  |  |  |
| NC_043001.1_32056050 |  |  | Intergenic region |  |  |  |
| NC_043003.1_89033637 |  |  | Intergenic region |  |  |  |
| NC_043005.1_32369504 |  |  | Intergenic region |  |  |  |
| NC_043009.1_7809789 |  |  | Intergenic region |  |  |  |
| NC_043010.1_27348758 |  |  | Intergenic region |  |  |  |
| NC_043015.1_44842899 |  |  | Intergenic region |  |  |  |
| NW_021831016.1_803 |  |  | Intergenic region |  |  |  |
| NW_021832251.1_41306 |  |  | Intergenic region |  |  |  |

**References**

Eirin-Lopez, J. M., & Putnam, H. M. (2019). Marine Environmental Epigenetics. *Annual Review of Marine Science*, *11*(1), 335–368. https://doi.org/10.1146/annurev-marine-010318-095114

Fellous, A., Wegner, K. M., John, U., Mark, F. C., & Shama, L. N. S. (2022). Windows of opportunity: Ocean warming shapes temperature‐sensitive epigenetic reprogramming and gene expression across gametogenesis and embryogenesis in marine stickleback. *Global Change Biology*, *28*(1), 54–71. https://doi.org/10.1111/gcb.15942

Pankov, K. V., McArthur, A. G., Gold, D. A., Nelson, D. R., Goldstone, J. V., & Wilson, J. Y. (2021). The cytochrome P450 (CYP) superfamily in cnidarians. *Scientific Reports*, *11*(1), 9834. https://doi.org/10.1038/s41598-021-88700-y

Pérez‐Portela, R., Riesgo, A., Wangensteen, O. S., Palacín, C., & Turon, X. (2020). Enjoying the warming Mediterranean: Transcriptomic responses to temperature changes of a thermophilous keystone species in benthic communities. *Molecular Ecology*, *29*(17), 3299–3315. https://doi.org/10.1111/mec.15564

Rosic, N. N., Pernice, M., Dunn, S., Dove, S., & Hoegh-Guldberg, O. (2010). Differential Regulation by Heat Stress of Novel Cytochrome P450 Genes from the Dinoflagellate Symbionts of Reef-Building Corals. *Applied and Environmental Microbiology*, *76*(9), 2823–2829. https://doi.org/10.1128/AEM.02984-09

Wang, W., Zheng, H., Wen, J., Xu, K., Xu, Y., Ji, D., Chen, C., & Xie, C. (2022). Early signaling events in the heat stress response of Pyropia haitanensis revealed by phosphoproteomic and lipidomic analyses. *Algal Research*, *67*, 102837. https://doi.org/10.1016/j.algal.2022.102837

Xu, D., & Wang, X. (2019). Lysine Acetylation is an Important Post-Translational Modification that Modulates Heat Shock Response in the Sea Cucumber Apostichopus japonicus. *International Journal of Molecular Sciences*, *20*(18), 4423. https://doi.org/10.3390/ijms20184423
