# Supplementary material for: Admixture and Selection Driven by El Niño–Southern Oscillation Events Shape the Genetic Structure of Octopus mimus–O. hubbsorum Complex Across the Humboldt and South Equatorial Current Transition Zone
Source: Ecol Evol. 2026 Sep 2;16(9):e74222. doi: 10.1002/ece3.74222 (PMC13539069; doi:10.1002/ece3.74222)
Supplement: Supplementary file 2 [file ECE3-16-e74222-s002.docx]

**Table S1.** Sample database with their respective location and sampling date. North, south (HCLME), transition zone (TZ).

| **Sample** | **Locality** | **Zone** | **Assigned population** | **Collection year** | **Mitochondrial genome accession Number** |
| --- | --- | --- | --- | --- | --- |
| Mex1 | *Mexico* | North | *O. hubbsorum* | 2024 | PX980416 |
| Mex2 | *Mexico* | North | *O. hubbsorum* | 2024 | PX980442 |
| Mex3 | *Mexico* | North | *O. hubbsorum* | 2024 | PX980438 |
| Mex4 | *Mexico* | North | *O. hubbsorum* | 2024 | PX980430 |
| Mex5 | *Mexico* | North | *O. hubbsorum* | 2024 | PX980448 |
| Mex6 | *Mexico* | North | *O. hubbsorum* | 2024 | PX980418 |
| Mex7 | *Mexico* | North | *O. hubbsorum* | 2024 | PX980441 |
| Mex8 | *Mexico* | North | *O. hubbsorum* | 2024 | PX980426 |
| Mex9 | *Mexico* | North | *O. hubbsorum* | 2024 | PX980419 |
| Mex10 | *Mexico* | North | *O. hubbsorum* | 2024 | PX980450 |
| OmTA1 | *Zorritos* | North | *O. hubbsorum* | 2014 | PX980444 |
| OmTA2 | *Zorritos* | North | *O. hubbsorum* | 2014 | PX980434 |
| OmTC1 | *Punta Sal* | North | *O. hubbsorum* | 2002 | PX980415 |
| OmTC2 | *Punta Sal* | North | *O. hubbsorum* | 2002 | PX980427 |
| OmTC3 | *Punta Sal* | North | *O. hubbsorum* | 2002 | PX980435 |
| OmTC4 | *Punta Sal* | North | *O. hubbsorum* | 2002 | PX980421 |
| OmPO1 | *Los Órganos* | North | *O. mimus* | 2002 | PX980428 |
| OmPO2 | *Los Órganos* | North | *O. hubbsorum* | 2002 | PX980429 |
| OmPO3 | *Los Órganos* | North | *O. hubbsorum* | 2002 | PX980443 |
| OmPO4 | *Los Órganos* | North | *O. mimus* | 2002 | PX980436 |
| CCM014 | *Los Órganos* | North | Admixed-Warm | 2020 | PQ040603 |
| APN45 | *El Ñuro* | North | Admixed-Warm | 2019 | PQ043215 |
| APN46 | *El Ñuro* | North | Admixed-Warm | 2019 | PQ043216 |
| APN47 | *El Ñuro* | North | Admixed-Warm | 2019 | PQ043217 |
| APN48 | *El Ñuro* | North | Admixed-Warm | 2019 | PQ043218 |
| APN49 | *El Ñuro* | North | Admixed-Warm | 2019 | PQ043219 |
| APN50 | *El Ñuro* | North | Admixed-Warm | 2019 | PQ043220 |
| OmS1 | *Sechura* | TZ | *O. mimus* | 2014 | PX980424 |
| OmS2 | *Sechura* | TZ | *O. hubbsorum* | 2014 | PX980417 |
| OmS3 | *Sechura* | TZ | *O. mimus* | 2014 | PX980449 |
| OmS4 | *Sechura* | TZ | *O. mimus* | 2014 | PX980425 |
| OmLT1 | *Islas Lobos de Tierra* | TZ | *O. mimus* | 2014 | PX980413 |
| OmLT2 | *Islas Lobos de Tierra* | TZ | *O. mimus* | 2014 | PX980439 |
| OmLT3 | *Islas Lobos de Tierra* | TZ | *O. mimus* | 2014 | PX980440 |
| OmLT4 | *Islas Lobos de Tierra* | TZ | *O. hubbsorum* | 2014 | PX980437 |
| EVO011 | *Islas Lobos de Afuera* | TZ | Admixed-Cold | 2018 | PQ040604 |
| VOE008 | *Islas Lobos de Afuera* | TZ | Admixed-Cold | 2018 | PQ043228 |
| VOE009 | *Islas Lobos de Afuera* | TZ | Admixed-Cold | 2018 | PQ043229 |
| VOE012 | *Islas Lobos de Afuera* | TZ | Admixed-Warm | 2018 | PQ043230 |
| VOE013 | *Islas Lobos de Afuera* | TZ | Admixed-Cold | 2018 | PQ043231 |
| VOE018 | *Islas Lobos de Afuera* | TZ | *O. mimus* | 2018 | PQ040609 |
| VOE019 | *Islas Lobos de Afuera* | TZ | *O. mimus* | 2018 | PQ043232 |
| VOE020 | *Islas Lobos de Afuera* | TZ | *O. mimus* | 2018 | PQ043233 |
| VOE021 | *Islas Lobos de Afuera* | TZ | Admixed-Cold | 2018 | PQ043234 |
| VOE022 | *Islas Lobos de Afuera* | TZ | Admixed-Cold | 2018 | PQ043235 |
| VOE024 | *Islas Lobos de Afuera* | TZ | Admixed-Cold | 2018 | PQ040606 |
| EAC304 | *Islas Lobos de Afuera* | TZ | Admixed-Cold | 2018 | PQ043225 |
| EAC309 | *Islas Lobos de Afuera* | TZ | Admixed-Cold | 2018 | PQ043226 |
| EAC310 | *Islas Lobos de Afuera* | TZ | Admixed-Cold | 2018 | PQ040605 |
| M1 | *Huarmey* | HCLME | Admixed-Cold | 2021 | PQ040607 |
| M2 | *Huarmey* | HCLME | Admixed-Cold | 2021 | PQ040608 |
| G | *Huarmey* | HCLME | Admixed-Cold | 2021 | PQ043227 |
| 18 | *Huarmey* | HCLME | Admixed-Cold | 2021 | PQ043214 |
| OmC1 | *Callao* | HCLME | *O. mimus* | 2012 | PX980432 |
| OmC2 | *Callao* | HCLME | *O. mimus* | 2012 | PX980423 |
| OmC3 | *Callao* | HCLME | *O. mimus* | 2012 | PX980446 |
| OmP1 | *Ica* | HCLME | *O. mimus* | 2012 | PX980422 |
| OmP2 | *Ica* | HCLME | *O. mimus* | 2012 | PX980447 |
| OmP3 | *Ica* | HCLME | *O. mimus* | 2012 | PX980414 |
| OmM1 | *Arequipa* | HCLME | *O. mimus* | 2011 | PX980431 |
| OmM2 | *Arequipa* | HCLME | *O. mimus* | 2011 | PX980420 |
| OmM4 | *Arequipa* | HCLME | *O. mimus* | 2011 | PX980433 |
| OmM5 | *Arequipa* | HCLME | *O. mimus* | 2011 | PX980445 |
| COA157 | *Ilo* | HCLME | Admixed-Cold | 2020 | PQ043221 |
| COA158 | *Ilo* | HCLME | Admixed-Cold | 2020 | PQ043222 |
| COA159 | *Ilo* | HCLME | Admixed-Cold | 2020 | PQ043223 |
| COA160 | *Ilo* | HCLME | Admixed-Cold | 2020 | PQ043224 |
